# Nanoparticles Equipped with α2,8-Linked Sialic Acid Chains Inhibit the Release of Neutrophil Extracellular Traps

**DOI:** 10.3390/nano9040610

**Published:** 2019-04-12

**Authors:** Kim F. Bornhöfft, Torsten Viergutz, Andrea Kühnle, Sebastian P. Galuska

**Affiliations:** 1 Institute of Reproductive Biology, Leibniz Institute for Farm Animal Biology (FBN), Wilhelm-Stahl-Allee 2, 18196 Dummerstorf, Germany; bornhoefft@fbn-dummerstorf.de (K.F.B.); viergutz@fbn-dummerstorf.de (T.V.); kuehnle@fbn-dummerstorf.de (A.K.); 2 Faculty of Medicine, Institute of Biochemistry, Justus-Liebig-University, Friedrichstrasse 24, 35392 Giessen, Germany

**Keywords:** neutrophil, NETosis, reactive oxygen species (ROS), innate immunity, sialic acids, siglecs, polysaccharide

## Abstract

Neutrophils can combat the invasion of pathogens by the formation of neutrophil extracellular traps (NETs). The NET mechanism is not only an effective tool for combating pathogens, but is also associated with diseases. Therefore, NETs are a potential target for combating pathologies, such as cystic fibrosis and thrombosis. We investigated the potential of nanoparticles, which were modified with α2,8-linked sialic acid chains, to modulate NET release during phorbol myristate acetate stimulation. Interestingly, when these nanoparticles were applied, the formation of reactive oxygen species was partly inhibited and the release of NET was counteracted. However, although the release of NET fibers was prevented, the nuclei still lost their characteristic segmented structure and became swollen, indicating that only the release, and not complete activation was suppressed. Intriguingly, coincubation of α2,8-sialylated particles with free sialic acid chains prevented the outlined inhibitory effects. Thus, the sialic acid chains must be attached to a linker molecule to generate an active bioconjugate that is able to inhibit the release of NET.

## 1. Introduction

Neutrophil granulocytes are the most abundant leukocytes of the innate immune system representing the first line of defense against invading pathogens [1,2,3]. In the case of inflammation, these granulocytes exit the circulation system via trans-endothelial migration, a selectin-driven mechanism [4]. Arriving at the inflammation site, they release reactive oxygen species (ROS), as well as antimicrobial peptides and phagocytose pathogens, such as bacteria.

Moreover, in 2004, Brinkmann and colleagues discovered that neutrophils can undergo beneficial suicide, resulting in the release of neutrophil extracellular traps (NETs) [5]. The DNA fibers released are associated with numerous antimicrobial components [5,6,7,8]. The formation of NETs can be induced by pathogens, such as bacteria, fungi, and viruses, as well as by chemical stimuli, such as calcium ionophore A23187 or phorbol myristate acetate (PMA) [9,10,11,12]. When the formation of NETs is induced by PMA, calcium influx from the endoplasmic reticulum is induced and protein kinase C alpha (PKC) is activated. PKC initiates the activation of the Raf-MEK-ERK pathway. ERK1/2 seems to activate nicotinamide adenine dinucleotide phosphate (NADPH) oxidase by phosphorylation, contributing to the activation of the NADPH oxidase enzyme complex [4,9,13,14,15]. This complex produces ROS, which are enzymatically converted to hypochlorous acid (HOCl) by myeloperoxidase [4,9]. This leads to the transfer of neutrophil elastase to the nucleus, where neutrophil elastase degrades histones, such as histone H4, promoting the decondensation of DNA. During the last step, granular vesicles, in addition to plasma membranes, rupture and a mixture, which consists of antimicrobial biomolecules (e.g., myeloperoxidases, neutrophil elastases, lactoferrin, defensins, and cytotoxic histones) is released to combat the invading pathogens [9,16].

In addition to beneficial effects, the formation of NET is associated with diseases, such as infertility, small-vessel vasculitis, rheumatoid arthritis, preeclampsia, ulcerative colitis, Crohn’s disease, systemic lupus erythematous, thrombosis, and cystic fibrosis [9,17,18,19,20,21,22,23]. Thus, the inhibition of an exaggerated NET release is an opportunity to combat these pathologies.

In mammals, several biomolecules have the capacity to modulate immunological mechanisms. Since all our cells are surrounded by a glycocalyx, consisting of glycolipids and glycoproteins, glycan-dependent mechanisms frequently take place during processes of innate and adaptive immunity [24]. In mammals, glycans are often terminated with sialic acid residues [25]. These sialylated structures can be recognized by immune cells using, for example, sialic acid-binding immunoglobulin–like lectins (siglecs), which are important immunoregulatory elements in vertebrates [26,27,28,29]. Intriguingly, inhibitory siglecs can counteract kinase-dependent activation of immune cells by recruiting SHP1 and SHP2 [28,30]. For example, Varki and colleagues described that, in the bloodstream, siglec-9 on neutrophils inhibits neutrophil activation by binding sialylated glycoproteins on erythrocytes [31]. Thus, siglecs became a target for modulating immunological events [32]. One possibility is to decorate nanoparticles with sialylated glycans. For instance, Spence et al. showed that nanoparticles coated with dimers of α2,8-linked sialic acid residues, which are the ligand for murine siglec-E, decreased inflammation driven by lipopolysaccharide (LPS) in murine macrophages [33]. Human neutrophils are known to express siglec-3, -5, -9, and -14, and interestingly, siglec-5 is able to recognize α2,8-linked sialic acid residues, such as siglec-E in mice [34,35,36].

Thus, we hypothesized that α2,8-linked sialic acid residues can be used to modulate the activation of neutrophils and NETosis. The obtained results demonstrate that the release of NETs is inhibited by the application of particles containing α2,8-linked sialic acid chains, indicating that α2,8-sialylated nanoparticles are a tool for manipulating the formation of NET.

## 2. Materials and Methods

### 2.1. Materials

Endoneuraminidase (endoN) was kindly provided by Martina Mühlenhoff (Medizinische Hochschule, Hannover, Germany) [37]. All reagents used were of analytical grade.

### 2.2. Human Neutrophils

All volunteers provided written informed consent and all samples were anonymized. The use of human neutrophils was approved by the local ethics office of the University of Giessen, School of Medicine (05/00).

### 2.3. Digestion and Fractionation of Sialic Acid Chains

10 mg colominic acid (Gerbu, Heidelberg, Germany) was digested via the use of endoN (4.46 µg mL^−1^, 2 h, 37 °C, 250 rpm). Resulting cleavage products were separated and collected according to the degree of polymerization (DP) by anion exchange chromatography, as described earlier, to get sialic acid chains with chain lengths consisting of <9 *N*-acetylneuraminic acid (Neu5Ac) residues [38,39,40]. The retention time of specific sialic acid chains was determined by mildly labeling 10 mg colominic acid with 1,2-diamino-4,5-methylenedioxybenzene (DMB) (Dojindo, Kumamoto, Japan) under the following conditions: A total of 10 mg colominic acid was dissolved in 200 μL DMB reaction buffer (9 mM sodium hydrosulfite, 0.5 M β-mercaptoethanol, 20 mM trifluoroacetic acid (TFA), and 1.35 M DMB) and 200 µL MilliQ water, incubated overnight at 11 °C, and stopped by the addition of 100 μL 1 M NaOH [39,41,42,43]. The different chain lengths were separated with a DNAPac PAc-100 column (22 mm × 250 mm; 13 μm; Thermo Fisher Scientific, Waltham, MA, USA) using HPLC system (Smartline System, Knauer, Berlin, Germany). MilliQ water (E1) and 2 M ammonium acetate buffer (E2) were used as eluents at a flow rate of 2.5 mL min^–1^. The following gradient was used for the separation, 0 min = 0% (*v*/*v*) E2, 20 min = 26% (*v/v*) E2, 30 min = 34% (*v*/*v*) E2, 45 min = 38% (*v*/*v*) E2, 85 min = 88% (*v*/*v*) E2, 110 min = 100% (*v*/*v*) E2, and 141 min = 0% (*v*/*v*) E2. DMB-labeled sialic acid chains were detected using a fluorescence detector at 372 nm for excitation and 456 nm for emission. In order to proof the degree of polymerization, aliquots of dried samples were mildly DMB labeled, as described above. Sample separation took place on an analytical DNAPac PAc-100 column (4 mm × 250 mm; 13 μm; Thermo Fisher Scientific). MilliQ water (E1) and 2 M ammonium acetate buffer (E2) were used as eluents at a flow rate of 1 mL min^–1^ using the following gradient, 0 min = 100% (*v*/*v*) E1, 5 min = 100% (*v*/*v*) E1, 15 min = 92% (*v*/*v*) E1, 20 min = 89% (*v*/*v*) E1, 30 min = 86% (*v*/*v*) E1, 55 min = 84% (*v*/*v*) E1, 100 min = 80% (*v*/*v*) E1, and 130 min = 77% (*v*/*v*) E1.

### 2.4. Quantification of Sialic Acids

To quantify the amount of N-acetylneuraminic acid, sample aliquots were hydrolyzed with 0.2 N TFA for 4 h at 80 °C. Dried samples, as well as appropriate standards, were then DMB-labeled under the following conditions: 40 µL DMB reagent + 40 µL MilliQ water, 2 h, 55 °C, 350 rpm. The labeling was stopped by the addition of 20 µL 0.2 M NaOH [44,45]. The quantification was performed with a LiCroCart 250-2 Merck and a SuperSpher 100 RP-C18 column, as described previously [44,45,46,47].

### 2.5. Coupling of Sialic Acids on Latex Beads

The separated sialic acid chains, as well as Neu5Ac (sialic acid monomers; monoSia) (Carbosynth, Compton, UK), were used for coupling reaction. Aliphatic amine latex particles (1% *w/v*, 0.1 µm; Thermo Fisher Scientific) were washed twice prior usage. A total of 150 µL latex particles were resuspended in 123 µL PBS and homogenization was performed by using ultrasonic. A total of 200 µg of dried sugars were dissolved in 25 µL PBS and then added to the latex particles. A total of 1.5 µL 5 M NaCNBH_3_ was added and the coupling reaction took place for 2 h at 65 °C and 250 rpm. After this, sialic acid–coupled particles were washed twice and resuspended in 100 µL PBS. An aliquot was taken to quantify the coupling reaction, as described in the previous chapter. The used nanoparticles showed no cytotoxic characteristics as tested previously in [40]. Regarding the stability of the sialylated particles, it should be noted that the sialylation status is reduced by 30% when stored at 8 °C for one month, resulting in a decreased activity.

### 2.6. Isolation of Human Neutrophils

Human neutrophils were isolated as described previously by Saffarzadeh et al. [48]. Therefore, a density gradient using a Histopaque-1077 and a Histopaque-1119 was applied at 37 °C and 700 × g for 30 min. The neutrophil containing layer was washed with PBS. After erylysis (lysis buffer: pH 7.5; 0.15 M NH_4_Cl, 0.1 mM EDTA, 1 mM KHCO_3_) cells were washed with PBS. The erylysis was performed two times. As a final step, the received pellet was washed and resuspended in RPMI 1640 (Thermo Fisher Scientific) with 1% penicillin/streptomycin (PenStrep; Thermo Fisher Scientific) and 1% fetal bovine serum (FBS; Thermo Fisher Scientific). A total of 30,000 cells/well were dissolved in RPMI 1640 with 1% PenStrep, and 1% FBS and incubated 1 h at 37 °C and 5% CO_2_ before the NETs were induced.

### 2.7. NETosis Stimulation and Inhibition Assay

The stimulation of neutrophils with 20 nM PMA took place in Poly-l-Lysine coated 12-well chamber slides for 2 h/4 h at 37 °C and 5% CO_2_. To inhibit NETosis cells were incubated with PMA and a final concentration of 10 µg mL^−1^ sialic acid was coupled on latex particles. Coincubations of α2,8-sialylated particles with 30 µg mL^−1^ free sialic acid chains of an inherent chain length were performed under equal conditions.

### 2.8. Immunofluorescence Staining

After 2 h or 4 h of PMA stimulation cells were fixed with 4% paraformaldehyde (PFA) for 30 min at 4 °C. After washing, cells were incubated with 0.5% Triton X-100 for 1 min, followed by three further washing steps. Blocking was performed for 30 min at 37 °C with 2% IgG free bovine serum albumin (BSA) (Carl Roth, Karlsruhe, Germany) and anti-Neutrophil Elastase (Abcam, Cambridge, UK) was incubated overnight at 4 °C. After further washing steps, the secondary antibody (FITC anti-rabbit, 1 h, room temperature (RT)) was added before the nuclei staining with 4′,6-Diamidin-2-phenylindol (DAPI) was done (Carl Roth, 1 µg mL^–1^). Fixation was performed with 2% PFA for 20 min at RT. Samples were then mounted and analyzed using fluorescence microscopy (Carl Zeiss confocal laser scanning microscope LSM 800). The determination of the NET area per cell was based on the release of decondensed DNA fibers during NETosis (DAPI staining) and was performed using Cellprofiler 2.2.0. Three pictures were randomly taken of each biological sample.

### 2.9. Measuring of the Production of Reactive Oxygen Species via Dihydrorhodamine 123 (DHR)

Since the production of reactive oxygen species reaches its maximum after 30 min of PMA incubation [49], 35,000 cells/well were seeded and DHR (Thermo Fisher Scientific) was coincubated with PMA with a final concentration of 1.445 µM diluted in RPMI 1640, 1% FBS, and 1% PenStrep for 30 min at 37 °C and 5% carbon dioxide. Measurement was performed with a Gallios, Beckman Coulter, at an excitation of 488 nm and with the emission at 525 nm ± 25 nm (argon laser).

### 2.10. Determination of the Membrane Potential with Bis-(1,3-Dibutylbarbituric Acid)trimethine Oxonol (DiBAC_4_)

The determination of the membrane potential was performed with DiBAC_4_. (Thermo Fisher Scientific). Therefore, 35,000 cells/well were seeded and NETosis was induced with 20 nM PMA for 3.5 h and cells were further incubated 30 min with 250 nM DiBAC_4_. Measuring took place with a Gallios, Beckman Coulter, at an excitation of 488 nm and with the emission at 525 nm ± 25 nm (argon laser), as described previously by Löhrke et al. [50].

### 2.11. NETose-Microscopy of Living Cells

After the isolation of human neutrophils, 50,000 cells/well were seeded in a Poly-l-Lysine -coated 12-well chamber slide. To de-stress cells from the isolation procedure, the following step was an incubation in RPMI 1640 for 1 h at 37 °C and 5% carbon dioxide. Afterwards, the cells were washed twice with RPMI 1640 and stained with 1 µg mL^–1^ DAPI (Carl Roth) for 30 min before a further washing step was done. To stain the membrane, Deep Red Cell Membrane Stain (1:1000; Thermo Fisher Scientific) was added 15 min before NETosis induction, with 50 nM PMA. Pictures were taken at an interval of 2 per min.

### 2.12. Determination of Membrane Integrity via Propidium Iodide (PI) Staining

Membrane integrity was analyzed using PI (Miltenyi Biotec, Bergisch Gladbach, Germany; Annexin V-FITC Kit) in a final concentration of 1 µg mL^–1^. After removing PI, nuclei were stained with Hoechst (Sigma Aldrich, St. Louis, MO, USA) in a final concentration of 1 µg mL^–1^. Cells were directly analyzed using fluorescence microscopy (Carl Zeiss confocal laser scanning microscope LSM 800). Three pictures were randomly taken from three independently performed experiments. The total cell number and PI positive cells were counted.

### 2.13. Statistical Analysis

Data sets were analyzed with Graph Pad Prism 7.0 software using ANOVA and a multiple-comparison Tukey test or a *t*-test with Welch’s correction, when the calculated values passed the Shapiro–Wilk normality test. Otherwise, the Kruskal–Wallis test in combination with Dunn’s test for multiple comparisons was applied. Differences were considered statistically significant at *p* ≤ 0.05. Statistically significant differences are given the labels * *p* ≤ 0.05; ** *p* ≤ 0.01; *** *p* ≤ 0.001; and **** *p* ≤ 0.0001.

## 3. Results

### 3.1. α2,8-Sialylated Particles Decrease the Production of Reactive Oxygen Species and Membrane Depolarization

**Figure 1:**
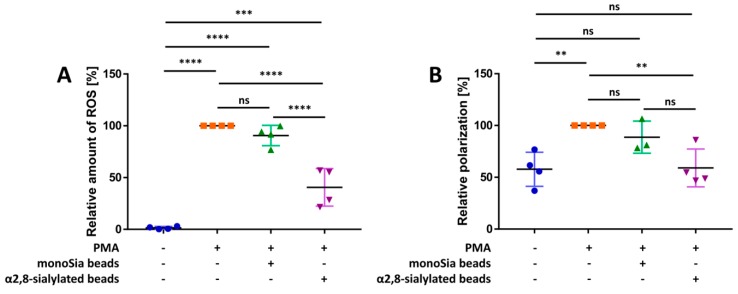
α2,8-sialylated particles reduce the production of ROS and the depolarization of the membrane when neutrophils are stimulated with PMA. (**A**) Scatter plot of the relative amount of ROS (%) produced after 30 min of stimulation with PMA. The determination of the relative amount of ROS (%) was performed using DHR. The values for PMA-treated cells were set to 100%. Four independent experiments were performed. (**B**) Scatter plot of the polarization status of the membrane. Determination of membrane depolarization was performed using DiBAC_4_. The emitted fluorescence of the PMA-induced cells was set to 100%. Mean values (*n* ≥ 3) and standard deviations are displayed in the diagrams. ANOVA and a multiple-comparison Tukey test were applied. Statistically significant differences are given as follows: ns, not significant, ** *p* ≤ 0.01; *** *p* ≤ 0.001; and **** *p* ≤ 0.0001.

NADPH oxidase represents a key enzyme in the signaling cascade during the formation of NET induced by PMA [51]. To test whether nanoparticles containing α2,8-linked sialic acid chains modulate the activation of neutrophils, ROS production was analyzed. To quantify ROS, DHR was used. In the presence of ROS, DHR is oxidized to cationic rhodamine 123, which exhibits green fluorescence. As shown in Figure 1A, in comparison to the neutrophils treated with PMA, unstimulated neutrophils assemble approximately 98% less ROS. When nanoparticles with sialic acid monomers were applied during the PMA stimulation, no effect on the PMA-induced ROS generation was observed. In contrast, the α2,8-sialylated nanoparticles statistically significantly decrease the ROS production that had been initiated by PMA. However, the production of ROS could not be completely abolished. Thus, chemically coupled α2,8-linked sialic acid chains seem to influence the activation of neutrophils.

The occurrence of membrane depolarization after activation of the NADPH oxidase complex was described in the 1980s [52,53,54]. Experiments focused on inhibition of NADPH oxidase complex–induced electron transport showed that the inhibition of electron transport leads to attenuated membrane depolarization [54]. In line with these experiments, neutrophils of patients with chronic granulomatous disease (CGD) showed no membrane depolarization after PMA stimulation, as this disease is characterized by an impaired NADPH oxidase complex [52,55,56]. Since α2,8-sialylated nanoparticles inhibit ROS production, we also examined whether membrane depolarization is influenced. To this end, the polarization status of the cells was determined with DiBAC_4_, a substance that can enter depolarized cells. Here, DiBAC_4_ can bind intracellular proteins exhibiting enhanced fluorescence and a red spectral shift. In Figure 1B, it is apparent that the stimulation of neutrophils with PMA results in cell membrane depolarization. Statistically significant differences between non-treated and stimulated cells were detected, which could not be influenced by monosialylated nanoparticles. However, in line with the ROS experiments, α2,8-sialylated nanoparticles inhibited membrane depolarization considerably.

In sum, the outlined results of the cell assays suggested that the essential steps during PMA stimulation, which trigger the formation of NET, are impaired by α2,8-sialylated nanoparticles.

### 3.2. The Swelling of Neutrophil Nuclei Is Not Influenced by α2,8-Sialylated Nanoparticles

The formation of ROS is directly linked to the translocation of neutrophil elastase into the nucleus because ROS serves as a substrate for myeloperoxidase. Subsequently, the increase in HOCl initiates the detachment of neutrophil elastase from the myeloperoxidase/neutrophil elastase complex, enabling the transfer of neutrophil elastase into the nucleus and triggering decondensation of DNA [4,9]. The result is that the nuclei lose their typical segmented structure and swell.

**Figure 2:**
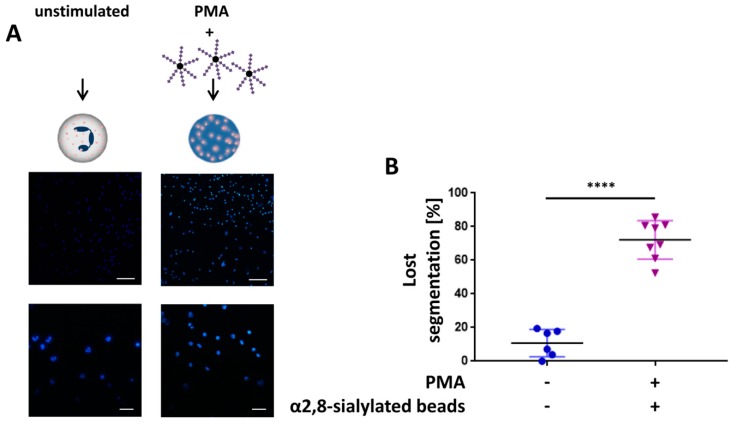
α2,8-sialylated nanoparticles do not prevent the loss of nucleus segmentation. (**A**) DNA was stained with DAPI (blue) in unstimulated neutrophils in addition to neutrophils, which were stimulated with PMA (20 nM PMA for 4 h) in the presence of α2,8-sialylated particles. Upper scale bar: 100 µm, lower scale bar: 20 µm. (**B**) Based on the fluorescence pictures, the total number of cells that lost their segmented structure was determined. Mean values (*n* ≥ 6) and standard deviations are displayed. The statistical significance was calculated with the *t*-test with Welch’s correction. The statistically significant difference is given as follows: **** *p* ≤ 0.0001.

When we compared the nuclear structure of unstimulated neutrophils with that of the activated neutrophils in the presence of α2,8-sialylated nanoparticles, we observed that nearly all nuclei lost their segmented structure and the nuclei were swollen 4 h after the beginning of the PMA stimulation (Figure 2). Thus, the results suggest that the decondensation of DNA could not be prevented.

The translocation of neutrophil elastase to the nuclei is an important step during the decondensation of DNA and the formation of NETs [16]. To test whether the localization of neutrophil elastase was also unchanged due to the application of α2,8-sialylated nanoparticles, the enzyme was visualized using a polyclonal Ab against neutrophil elastase. Based on published data concerning the time point of the translocation of neutrophil elastase into the nucleus, immunofluorescence staining of neutrophil elastases was performed 2 h after the PMA treatment [16].

**Figure 3:**
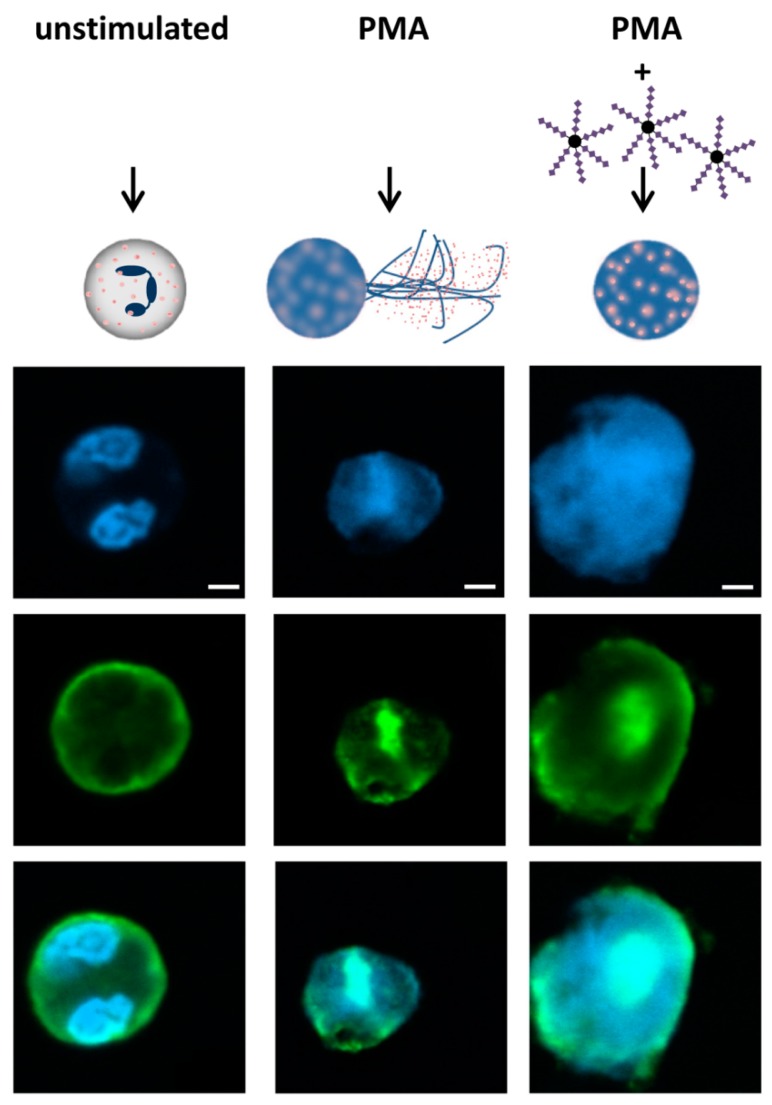
Neutrophil elastase also enters the nucleus during the application of α2,8-sialylated nanoparticles. Cells were incubated with 20 nM PMA for 2 h and were stained with a polyclonal antibody against neutrophil elastase (FITC; green). DNA was visualized using DAPI (blue). Scale bar: 2 µm.

In contrast to the unstimulated neutrophils, neutrophil elastase was transferred into the nucleus after stimulation (Figure 3). In addition, the segmented structure of the nucleus was resolved. Comparable results were obtained using α2,8-sialylated particles. Thus, the reduction of ROS to approximately 40%, which was induced by α2,8-sialylated nanoparticles, does not prevent the transformation of the nuclei.

### 3.3. α2,8-Sialylated Particles Inhibit the Release of NET 

**Figure 4:**
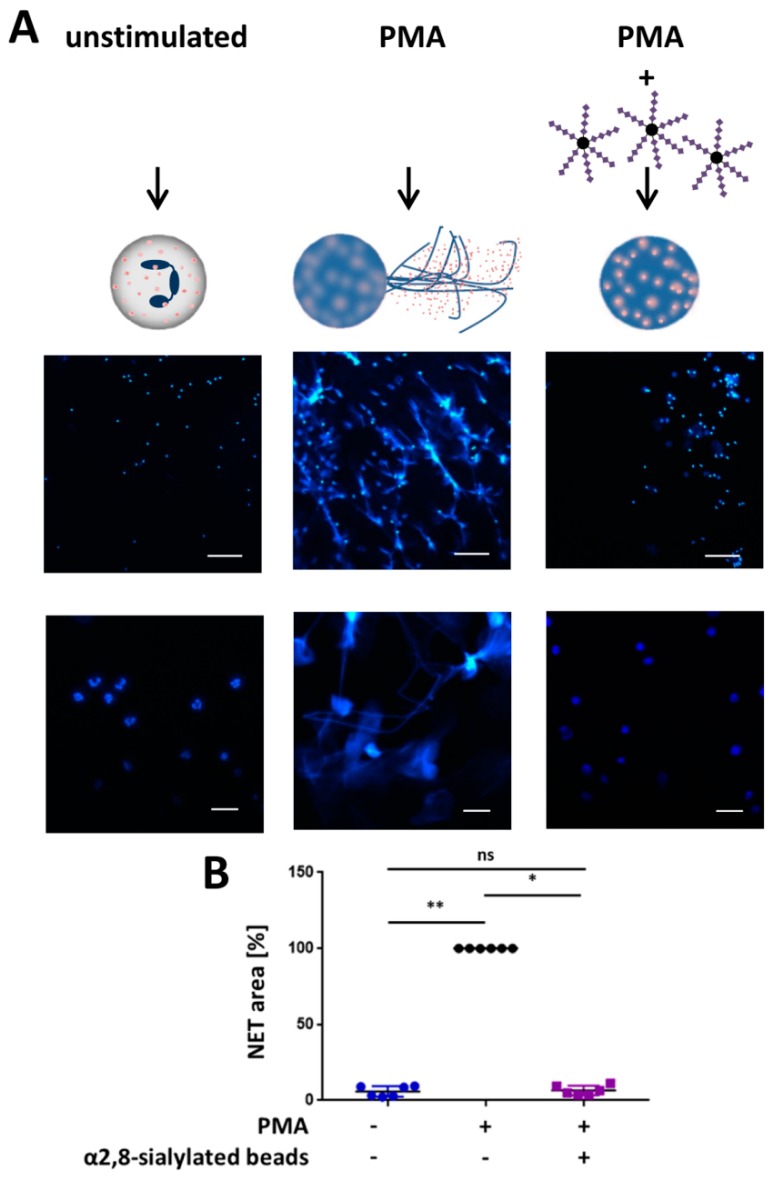
α2,8-sialylated particles inhibit NET release. (**A**) Fluorescence staining (DAPI; blue) of neutrophils without any stimulation of neutrophils, stimulated with 20 nM PMA for 4 h, and of neutrophils stimulated with 20 nM PMA coincubated with α2,8-sialylated particles for 4 h. Upper scale bar: 100 µm, lower scale bar: 20 µm. (**B**) Analysis of the NET area (%). The analysis was performed using Cell Profiler 2.2.0. The NETosis area (%) was calculated by determining the blue fluorescent areas (DAPI). PMA inducement was set to 100%. Mean values (*n* = 6) and standard deviations are displayed. The Kruskal–Wallis test and Dunn’s test for multiple comparisons were applied. Statistically significant differences are given as follows: ns, not significant, * *p* ≤ 0.05 and ** *p* ≤ 0.01.

In addition, the release of NET was examined. The stimulation of NETosis leads to the formation of the DNA meshwork, which can be visualized with DAPI. As shown in Figure 4A,B, 4 h after PMA stimulation, the expected NET filaments are formed. In contrast, the unstimulated neutrophils retained their shape, including their segmented nuclei. However, the application of α2,8-sialylated nanoparticles prevented NET release although the nuclei were swollen. The calculation of the NET area shows that NET expansion was statistically significantly reduced.

**Figure 5:**
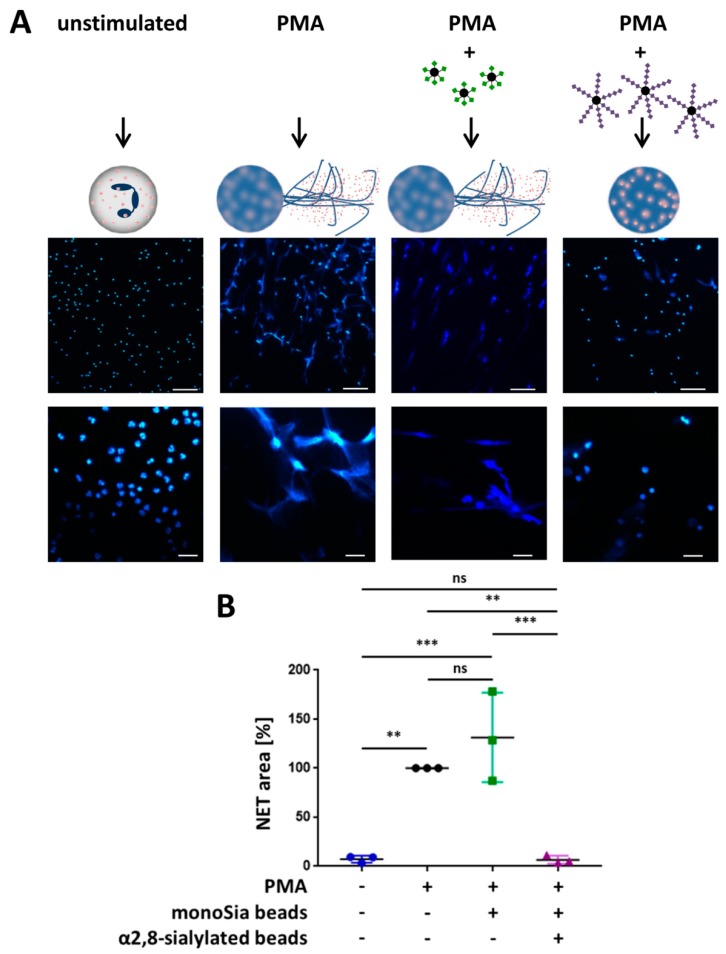
α2,8-sialylated particles inhibit NET release. (**A**) Fluorescence staining (DAPI; blue) of neutrophils without any stimulation of neutrophils, stimulated with 20 nM PMA for 4 h, and of neutrophils stimulated with 20 nM PMA coincubated with monosialylated particles or α2,8-sialylated particles for 4 h. Upper scale bar: 100 µm, lower scale bar: 20 µm. (**B**) Analysis of the NET area (%). The analysis was performed using Cell Profiler 2.2.0. The NETosis area (%) was calculated by determining the blue fluorescent areas (DAPI). PMA inducement was set to 100%. Mean values (*n* = 3) and standard deviations are displayed. ANOVA and multiple comparison Tukey test were applied. Significant differences are given as follows: ns, not significant, ** *p* ≤ 0.01 and *** *p* ≤ 0.001.

**Figure 6:**
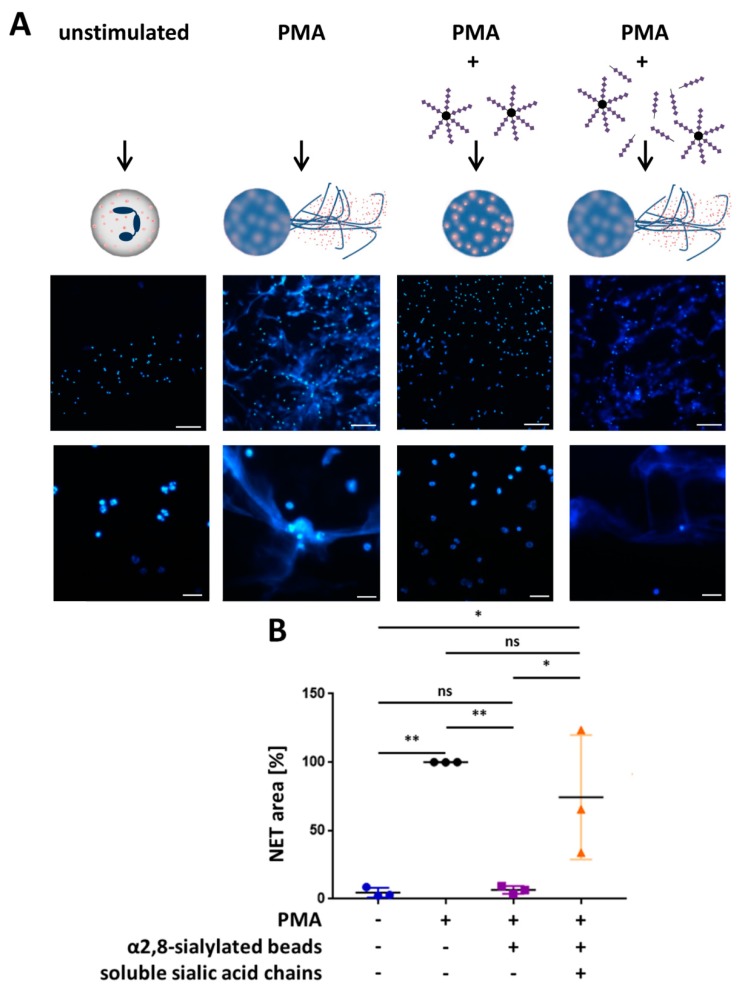
Free sialic acid chains prevent the outlined inhibitory effects of α2,8-sialylated particles. (**A**) Fluorescence staining (DAPI; blue) of isolated human neutrophils stimulated with 20 nM PMA and coincubated with α2,8-sialylated particles or free sialic acid chains in combination with α2,8-sialylated nanoparticles. Upper scale bar: 100 µm, lower scale bar: 20 µm. (**B**) Analysis of the NET area (%). Analysis was performed on three different experimental approaches using Cell Profiler 2.2.0. NETosis area (%) was calculated by determining the blue fluorescent areas (DAPI). PMA stimulation was set to 100%. Mean values and standard deviations are displayed in the diagrams. ANOVA and multiple comparison Tukey test were applied. Significant differences are given as follows: ns, not significant, * *p* ≤ 0.05 and ** *p* ≤ 0.01.

The same experimental test setup was also performed with monosialylated beads, which showed no impact on the activation by PMA (Figure 5). In addition, experiments were performed to investigate whether free sialic acid chains have the same effect or whether sialic acid chains have to be attached to a linker to inhibit the release of NETs. To this end, we used free sialic acid chains together with the α2,8-sialylated beads. Again, α2,8-sialylated nanoparticles had the capability to prevent NET release (Figure 6). However, this effect was inhibited by free sialic acid chains. Thus, a linker molecule is necessary to inhibit the release of NETs.

### 3.4. α2,8-Sialylated Particles Prevent Cell Membrane Perforation

**Figure 7:**
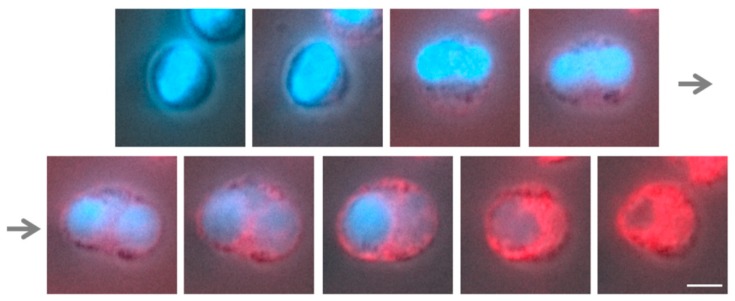
Release of NETs. NETosis stimulation was performed with 50 nM PMA. At the very beginning of PMA induction, a healthy cell with an intact nucleus is visible. Then DNA decondensation takes place, followed by disintegration of the nuclear membrane and a fusion of DNA with the released cytoplasmic proteins. Thus, at this time point, the cell dimension expands, leading to a rupture of the outer membrane, along with the release of the DNA-protein mixture into the fluid surrounding the cell. The empty cell membrane remains behind. The cell membrane (red) was stained with Deep Red and DNA (blue) with DAPI. Scale bar: 5 µm.

For the release of NETs, cell membrane rupture is necessary [9]. Using Deep Red Cell Membrane Staining, it is possible to visualize the cellular membrane during NETosis and its explosive bursts to release the DNA network (illustrated in Figure 7 and Supplemental 1; video). The resulting hole allowed the release of intracellular substances and the penetration of molecules into the cell. Interestingly, the intensity of the membrane staining became stronger during the alteration of the nuclear structure, indicating that the membrane properties of the neutrophils had changed before the release of NETs.

**Figure 8:**
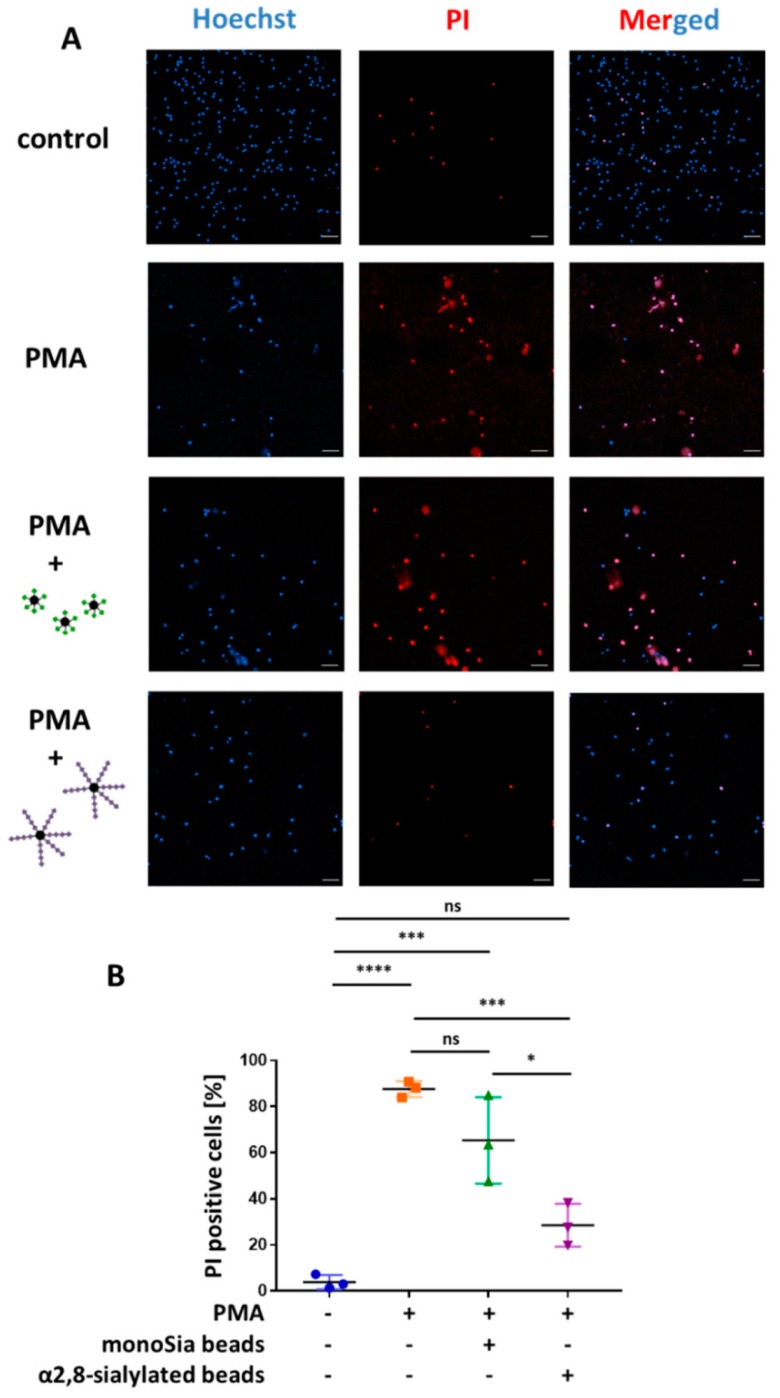
Coincubation of PMA-stimulated neutrophils with α2,8-sialylated particles significantly decreases the number of PI-positive cells. (**A**) Isolated neutrophils stimulated with 20 nM PMA and isolated neutrophils stimulated with 20 nM PMA coincubated with α2,8-sialylated particles or monosialylated particles were stained with PI (red) and Hoechst (blue) to determine membrane permeability. Unstimulated isolated neutrophils served as a control. Scale bars: 50 µm. (**B**) Based on the fluorescence images the total number of cells was determined and was set in relation to the number of PI-positive cells. Mean values (*n* = 3) and standard deviations are displayed in the diagrams. ANOVA and a multiple-comparison Tukey test were applied. Significant differences are given as follows: ns, not significant, * *p* ≤ 0.05; *** *p* ≤ 0.001; and **** *p* ≤ 0.0001.

In order to test whether the outer membranes were still intact we used PI, a substance that can pass only perforated, not intact, biomembranes. Once PI enters the cell, it interacts with DNA, resulting in a red fluorescent staining. The calculation was performed by counting the total number of cells compared to the number of PI-positive cells. The results indicate that, compared to untreated neutrophils, PMA led to an increasing number of perforated cells (Figure 8). Similar results were obtained when monosialylated beads were added. However, the application of α2,8-sialylated beads resulted in a significantly reduced number of PI-positive cells, demonstrating that α2,8-sialylated beads inhibit the perforation of the biomembrane, and thus, the release NET.

## 4. Discussion

In 2015, Spence et al. discovered that nanoparticles coated with the murine siglec-E ligand, α2,8-linked sialic acid residues, decrease macrophage-driven inflammation [33]. Based on this discovery, we decided to investigate the potential of α2,8-sialylated particles to inhibit the release of NETs. Interestingly, the application of α2,8-sialylated particles decreased the production of ROS in PMA-stimulated neutrophils, while monosialylated particles showed no effect. In line with the reduced ROS, depolarization was also inhibited by these sugar-coated nanoparticles. Since the activation of the NADPH oxidase complex includes PKC and the Raf-MEK-ERK pathway, resulting in the formation of ROS [14,57], and siglecs are known to inhibit the immune response by counteracting kinases via the recruitment of the phosphatases SHP1 and SHP2 [28,30], the involvement of siglecs may trigger these observations. As siglec-5 is known to prefer α2,8-linked sialic acids, decreased ROS production and depolarization might be the result of the interaction between α2,8-linked chains and siglec-5 of neutrophils [34,35,36]. However, the impaired ROS production and depolarization did not prevent swelling of the nuclei. This indirectly indicates that neutrophil elastases still translocate to the nuclei, because neutrophil elastase is necessary for decondensation of DNA and, therefore, for nuclei swelling as described previously [16].

Nevertheless, the release of NETs was retained and the α2,8-sialylated particles prevented the perforation of the outer membrane (Figure 8). Interestingly, a comparable effect was described for lactoferrin [58]. In line with α2,8-sialylated beads, elastase translocates to the nucleus in the presence of lactoferrin and nucleus swelling takes place. In addition, in the case of lactoferrin, the last step, the release of NET is inhibited. Okubo and colleagues observed that lactoferrin accumulates on the surface of neutrophils [58]. They suggested a physical blocking barrier, which is mediated via a “lactoferrin-shell”. The first released DNA aggregates with lactoferrin forming a plug, which prevents the release of NETs [58]. As recently shown, polysialic acid (polySia) can interact with lactoferrin and supports the effect of lactoferrin to inhibit NET release [59]. However, whether the membrane was still intact was not tested. 

In addition to lactoferrin, α2,8-linked polymers of sialic acids are known to induce membrane interactions, because these polymers can interact with phospholipids [60]. This interaction usually takes place in ordered regions of membranes, such as lipid rafts [61]. The staining of cell membranes with Deep Red during NETosis (Figure 7) suggested that fluid and dynamic processes occur in the biomembrane, which might mediate such an interaction. Thus, we suggest that the membrane is stabilized by the multivalency of α2,8-sialylated nanoparticles, which might act as “cross struts”. Furthermore, perhaps an interaction with membrane proteins, such as siglec-5, supports the stabilization of the cell membrane in a comparable fashion. These hypotheses are supported by the observation that free sialic acid chains inhibit the effects of the α2,8-sialylated nanoparticles.

However, in contrast to lactoferrin, α2,8-sialylated beads influence ROS production [58], which is essential for the formation of NETs induced by PMA. The observed inhibition of ROS formation might be the result of an activation of siglec-5 by the α2,8-sialylated beads. The lower ROS content might additionally reduce the dynamic of the neutrophil burst. Presumably, the combination of these two effects (the “cross struts” effect and lower ROS levels) leads to stabilization of the biomembrane, which prevents the cell membrane from rupturing.

Although the exact mechanism of action has been unknown until now, α2,8-sialylated nanoparticles are a promising option for modulating NETosis during NET-related pathologies. Furthermore, for application as a pharmacological tool, the α2,8-sialylated nanoparticles must be tested in different in vivo models where NETs are the main cause that induces pathology.

## Supplementary Materials

The following are available online at https://www.mdpi.com/2079-4991/9/4/610/s1, Supplemental 1: Visualization of NETosis.
