# Supplementary material for: Nanoparticles Equipped with α2,8-Linked Sialic Acid Chains Inhibit the Release of Neutrophil Extracellular Traps
Source: Nanomaterials (Basel). 2019 Apr 12;9(4):610. doi: 10.3390/nano9040610 (PMC6523985; doi:10.3390/nano9040610)
Supplement: Supplementary file 1 [file nanomaterials-09-00610-s001.zip › nanomaterials-450886-SI.pptx]

## Slide 1
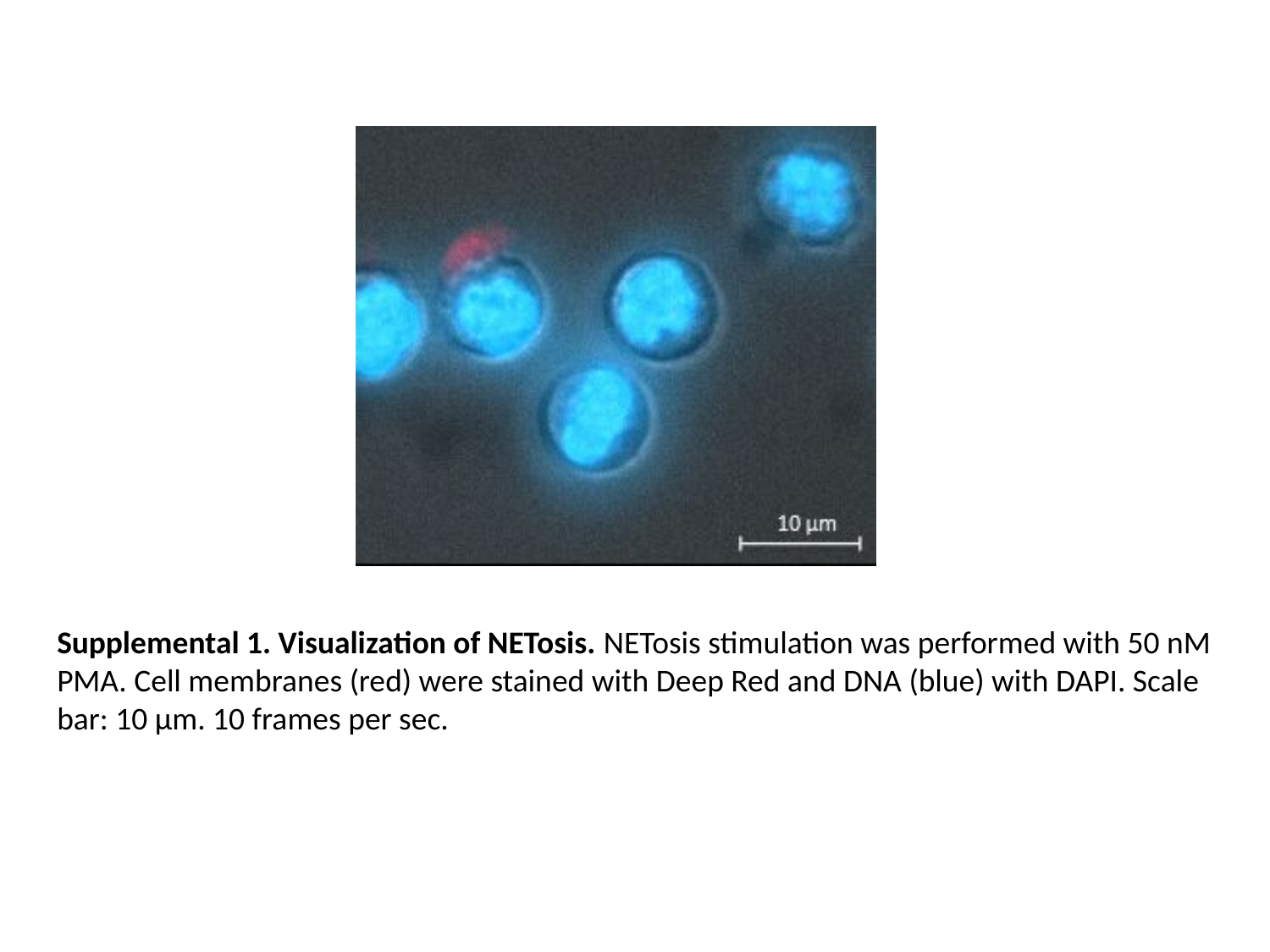

Supplemental 1. Visualization of NETosis. NETosis stimulation was performed with 50 nM PMA. Cell membranes (red) were stained with Deep Red and DNA (blue) with DAPI. Scale bar: 10 µm. 10 frames per sec.
